# Prevalence and clinical impact of iron deficiency and anaemia among outpatients with chronic heart failure: The PrEP Registry

**DOI:** 10.1007/s00392-016-1073-y

**Published:** 2017-02-22

**Authors:** Stephan von Haehling, Uwe Gremmler, Michael Krumm, Frank Mibach, Norbert Schön, Jens Taggeselle, Johannes B. Dahm, Christiane E. Angermann

**Affiliations:** 1 Department of Cardiology and Pneumology, University of Göttingen Medical School, Robert-Koch-Strasse 40, 37075 Göttingen, Germany; 2 MVZ Ambulantes kardiologisches Zentrum, Peine, Germany; 3 Kardiologische Praxis, Gesundheitszentrum Klosterforst, Itzehoe, Germany; 4 Kardiologisch-angiologische Praxis, Mühldorf am Inn, Germany; 5 Kardiologische Praxis, Markkleeberg, Germany; 6 Praxis und Klinik für Kardiologie und Angiologie, Herz- und Gefässzentrum, Krankenhaus Neu-Bethlehem, Göttingen, Germany; 7 Department of Medicine I, Comprehensive Heart Failure Center, University Hospital Würzburg, University of Würzburg, Würzburg, Germany

**Keywords:** Iron deficiency, Anaemia, Exercise capacity, Heart failure, Prevalence

## Abstract

**Background:**

Iron deficiency (ID) and anaemia are common in heart failure (HF). The prospective, observational PReP registry *(Prävalenz des Eisenmangels bei Patienten mit Herzinsuffizienz)* studied prevalence and clinical impact of ID and anaemia in HF outpatients attending cardiology practices in Germany.

**Methods and results:**

A total of 42 practices enrolled consecutive patients with chronic HF [left ventricular ejection fraction (LVEF) ≤45%]. ID was defined as serum ferritin <100 µg/l, or serum ferritin ≥100 µg/l/<300 µg/l plus transferrin saturation <20%, and anaemia as haemoglobin <13 g/dl (12 g/dl) in men (women). Exercise capacity was assessed using spiroergometry (69.4%) or 6-min walk test (30.4%). Amongst 1198 PReP-participants [69.0  ± 10.6 years, 25.3% female, New York Heart Association (NYHA) class 2.4  ± 0.5, LVEF 35.3 ± 7.2%], ID was found in 42.5% (previously unknown in all), and anaemia in 18.9% (previously known in 4.8%). ID was associated with female gender, lower body weight and haemoglobin, higher NYHA class and natriuretic peptide (NP) levels (all *p* < 0.05). ID was also more common in anaemic than non-anaemic patients (*p* < 0.0001), and 9.8% of PrEP-participants had both, ID and anaemia. On spiroergometry, ID independently predicted maximum exercise capacity even after multivariable adjustment, including anaemia (*p* = 0.0004). In all PrEP-participants, ID predicted reduced physical performance (adjusted for age, gender, anaemia, serum creatinine, C-reactive protein, LVEF, and NP level).

**Conclusions:**

Despite high prevalence, ID was previously unknown in all PrEP-participants, and anaemia was often unappreciated. Given the clinical relevance, treatability, and independent association with reduced physical performance, ID should be considered more in real-world ambulatory healthcare settings and ID-screening be advocated to cardiologists in such populations.

## Introduction

Besides anaemia, iron deficiency (ID) has more recently been recognized as a separate clinically relevant co-morbidity in patients with heart failure (HF) and other cardiovascular illnesses, with serious consequences for patient well-being and outcomes [1, 2]. The pivotal importance of iron is based on its essential role in oxygen transport, and its central role in processes maintaining cellular energy in high-energy demanding tissues like cardiac muscle [3–5].

ID can be classified into absolute ID, reflecting depleted iron stores, and functional ID, where iron delivery to target cells is hampered despite normal or overly abundant iron stores [6]. In healthy subjects, ID is generally diagnosed using a serum ferritin cut-off level <30 µg/l for absolute ID [7]. Because ferritin is also an acute phase reactant, pro-inflammatory activity increases its synthesis, thus rendering the diagnosis of ID more difficult in diseases associated with chronic subclinical inflammation. A ferritin cut-off value <100 µg/l is currently considered diagnostic for ID in patients with HF irrespective of transferrin saturation (TSAT). Alternatively, a cut-off value <300 µg/l together with a TSAT <20% may be used [8, 9]. Reduced TSAT is viewed as an indicator of insufficient iron availability for metabolizing cells [10].

Dependent on the various ID definitions, the prevalence of ID has been estimated between 13 and 34% with higher incidence rates among anaemic patients [11, 12]. Using the above diagnostic criteria, Jankowska et al. recently documented a 37% prevalence of ID among 546 patients with chronic systolic HF [13] and identified female gender, advanced disease (New York Heart Association class, and elevated plasma N-terminal pro-B-type natriuretic peptide (NT-proBNP) and serum high-sensitivity C-reactive protein levels as independent predictors of a higher likelihood of ID in a relatively young and predominantly male HF population. Little is known about the prevalence and clinical impact of ID with or without concurrent anaemia in a clinically stable, community-dwelling outpatient population with chronic HF. The prospective observational PReP registry *(Prävalenz des Eisenmangels bei Patienten mit Herzinsuffizienz)* was created to study prevalence and clinical impact of ID and anaemia in a real-world setting of ambulatory patients with HF.

## Methods

Between November 2010 and March 2012, ambulatory patients with chronic HF who presented at one of 42 office-based cardiologist practices in Germany were recruited into the prospective PrEP registry. All male and female patients aged 18 years and older were eligible, provided a left ventricular ejection fraction of 45% or less was documented on echocardiography during the enrolment visit and signs and symptoms of chronic heart failure were present. All patients gave written informed consent to the analysis of their pseudonymized clinical and laboratory data as part of the PReP registry. Practices were asked to include patients consecutively. None of the participating cardiologists reported any patients who *a priori* declined participation. However, the study protocol did not foresee entry of all screened patients into a screening and enrolment log sheet. Exclusion criteria were coronary interventions of any kind within the past 6 months and planned coronary interventions, evidence of acute or chronic infectious or inflammatory conditions from routine laboratory assessment, malignant disease or gastric or duodenal ulcer with or without active bleeding, and lack of written informed consent. The ethics committee of the Bavarian Chamber of Physicians, Munich, Germany, approved the study, and all patients provided written informed consent. The study was conducted in accordance with the Declaration of Helsinki.

A total of 1602 patients with chronic symptomatic HF were enrolled. Four hundred and four of these participants had to be excluded from the final data set and subsequent analysis due to protocol violations (particularly in one centre whose entire patient cohort had to be excluded because of insufficient or ambiguous data documentation) or because essential data were missing. Patient baseline assessment included a standardized HF history regarding HF aetiology (classified as ischaemic or non-ischaemic) and co-morbidities.

All patients underwent a standardized clinical evaluation, including physical examination, determination of NYHA class, determination of body weight, and determination of the heart rate from the electrocardiogram and of blood pressure by the Riva-Rocci method. In addition, patients underwent semiquantitative assessment of their volume status, in which peripheral oedema was classified as absent, mild, or significant. Blood samples were drawn from an antecubital vein in the morning for the assessment of a full blood count and clinical chemistry, including parameters of iron metabolism [serum ferritin, transferrin, and transferrin saturation (TSAT)] and kidney function (creatinine).

Renal dysfunction was diagnosed if the glomerular filtration rate was below 60 ml/min/1.73 m^2^, diabetes mellitus, if patients reported a history of diabetes or were on anti-diabetic drugs, and chronic obstructive pulmonary disease (COPD), if patients were on anti-obstructive pharmacotherapy or reported that COPD had been previously diagnosed. A history of depression was assumed if patients reported a previous respective physician diagnosis or were undergoing any specific antidepressant therapy. The diagnosis of restless legs was documented if patients reported that this condition had been previously diagnosed. Anaemia was defined according to World Health Organization criteria as haemoglobin level <12 g/dl in women and <13 g/dl in men [14]. ID was defined as serum ferritin <100 µg/l, or serum ferritin ≥100 µg/l and <300 µg/l with TSAT <20%. For natriuretic peptide assessment, either determination of B-type natriuretic peptide (BNP) or N-terminal-proBNP (NT-proBNP) was permitted according to local standards. Assessment of exercise capacity was performed either by spiroergometry testing or a 6-min corridor walk test, depending on local circumstances and availability. Exercise capacitly was categorized as reduced, if patients performed below the median of the respective test, i.e., below the median exercise capacity at spiroergometry or below the median walking distancene during the 6-min walk test, respectively.

### Statistical analyses

Continuous variables are given as means with standard deviations. Non-normally distributed variables (serum ferritin, serum creatinine, serum C-reactive protein, and natriuretic peptides) were log-transformed to achieve normal distribution before analysis. Student’s *t* test and analysis of variance (ANOVA) with Fisher’s post-hoc test were used to test for between-group differences. Categorical variables were expressed as numbers with percentages and the Chi-square test was used to test for inter-group proportion differences. Simple regression was used to analyse the first-line associations between continuous variables.

Univariate and multivariate logistic regression models were used to identify clinical determinants of exercise capacity in patients with chronic HF. The analyses included continuous and dichotomized variables of the parameters age, gender, NYHA class, LVEF, natriuretic peptide levels, the presence of anaemia, and the presence of ID. All statistical analyses were performed using StatView version 5.0 for Mac (Abacus Concepts, Berkeley, California). All tests were two-sided. *P* values of <0.05 were considered statistically significant.

## Results

The final data set comprised 1198 patients, 25.7% of whom were female. Baseline characteristics are given in Table 1. Patients were elderly, with a mean age of 69.0  ± 10.6 years. HF aetiology was predominantly ischaemic (62.4%). The NYHA distribution was *n* = 730 (60.9%) for NYHA class II, *n* = 457 (38.1%) for class III, and *n* = 11 (0.01%) for class IV. HF medications included angiotensin-converting enzyme inhibitors (68.6%), angiotensin receptor blockers (24.6%), beta-blockers (86.1%), mineralocorticoid receptor antagonists (39.1%), and digitalis (18.4%). A total of 87.9% of all patients received either an angiotensin-converting enzyme inhibitor or an angiotensin receptor blocker, and 64 patients (5.3%) received both. Diuretics were prescribed in 76.3% of the patients. A total of 864 patients (69.6%) underwent spiroergometry and the remaining 334 patients (30.4%) completed a 6-min walk test.

**Table 1:** Baseline characteristics of the total study population and of patients with versus without iron deficiency

	All patients	Patients with iron deficiency (*n* = 509)	Patients without iron deficiency (*n* = 689)	P*
Demographics				
Age (years)	69.0 ± 10.6	69.0 ± 11.2	69.0 ± 10.1	0.99
Male gender (%)	74.3	66.4	80.1	<0.0001
Body weight (kg)	84.9 ± 17.9	83.3 ± 18.4	86.0 ± 17.5	0.01
Heart rate (min^−1^)	71.7 ± 13.0	72.4 ± 12.8	71.3 ± 13.1	0.15
Systolic BP (mmHg)	129 ± 20	129 ± 21	129 ± 19	0.64
Diastolic BP (mmHg)	78 ± 11	77 ± 11	78 ± 11	0.46
HF characteristics				
Ischaemic aetioogy (%)	62.4	64.4	60.8	0.20
NYHA class	2.4 ± 0.5	2.5 ± 0.5	2.4 ± 0.5	0.0003
LVEF (%)	35.3 ± 7.2	35.0 ± 7.5	35.6 ± 7.0	0.17
Laboratory assessments				
Haemoglobin (g/dl)	13.9 ± 1.6	13.6 ± 1.7	14.2 ± 1.5	<0.0001
Leukocytes (µl^− 1^)	7617 ± 3793	7757 ± 3491	7505 ± 4015	0.35
Platelets (µl^− 1^)	214,690 ± 64,043	223,440 ± 68,593	208,227 ± 59,695	<0.0001
Serum ferritin (µg/l)	196 ± 197	77.1 ± 53.6	285 ± 218	<0.0001
TSAT (%)	24.2 ± 9.3	19.1 ± 8.6	28.2 ± 7.7	<0.0001
Serum creatinine (mg/dl)	1.2 ± 0.6	1.2 ± 0.4	1.2 ± 0.6	0.05
CRP (mg/dl)	1.9 ± 5.7	2.0 ± 4.3	1.8 ± 6.5	0.004
BNP (pg/ml) [*n* = 392]	346 ± 623	442 ± 675	282 ± 577	0.01
NT-proBNP (pg/ml) [*n* = 810]	1449 ± 1906	1690 ± 2252	1263 ± 1567	0.002
Co-morbidities and risk factors				
Anaemia (%)^b^	18.9	23.0	15.8	0.02
Hypertension (%)^a^	68.7	69.2	68.4	0.09
Renal dsyfunction (%)^c^	22.7	23.0	22.5	0.84
COPD (%)**	8.9	10.6	7.7	0.08
Diabetes mellitus (%)^d^	30.0	32.4	28.2	0.11
History of depression (%)^e^	5.7	8.0	3.9	0.002
Restless legs syndrome (%)^f^	2.6	3.7	1.7	0.03

Values are mean ± standard deviation or number of patients (%), apart from biomarker values which are reported as median [interquartile-range]

*BP* Blood pressure, *BNP* B-type natriuretic peptide, *COPD* chronic obstructive pulmonary disease, *LVEF* left ventricular ejection fraction, *NT-proBNP* N-terminal pro-B-type natriuretic peptide, *NYHA* New York heart association, *TSAT* transferrin saturation

*Student’s *t* test and analysis of variance (ANOVA) with Fisher’s post-hoc test

**COPD chronic obstructive pulmonary disease, either requiring bronchiolytic treatment or patient-reported previous diagnosis

^a^Hypertension: sitting blood pressure >140/90 mmHg or history of hypertension

^b^Anaemia: haemoglobin <12 mg/dl (females) or < 13 mg/dl (males)

^c^Renal dysfunction: estimated glomerular filtration rate <60 mL/min/1.73 m^2^.

^d^Diabetes mellitus: history of diabetes mellitus

^e^History of depression: either patient-reported physician diagnosis of depression or on antidepressant therapy

^f^Restless leg syndrome: patient-reported previous diagnosis

### Prevalence of iron deficiency

ID was present in 509 (42.5%) of the PrEP-participants and not previously known in any of them. None of the patients had a history of iron supplementation of any kind. Compared with PrEP-participants without ID, these patients had lower body weight (*p* = 0.01), haemoglobin levels (<0.0001) and mean corpuscular haemoglobin volume (*p* < 0.0001), and higher platelet count (*p* < 0.0001), NYHA class (*p* = 0.0003), and B-type natriuretic peptide (BNP) (*p* = 0.01) or N-terminal pro-B-type natriuretic peptide (NT-proBNP) values (*p* = 0.0015, Table 1). By contrast, no differences were recorded for age, resting heart rate, blood pressure, LVEF, leukocyte count, C-reactive protein, or serum creatinine (all *p* > 0.05).

Among patients who reported to suffer from restless legs syndrome and those with a history of depression, ID was significantly more prevalent than in the group without ID (Table 1, *p* = 0.03 and *p* = 0.002, respectively). No such differences were found regarding hypertension, diabetes, renal dysfunction, or chronic obstructive pulmonary disease.

### Prevalence of anaemia

Two hundred and twenty six (18.9%) of the PrEP-participants presented with anaemia. Despite high prevalence, this co-morbidity was known prior to enrolment only in 4.8%. None of the patients had received erythropoietin or blood transfusion for the treatment of anaemia. Anaemic patients were more likely to have concomitant ID than non-anaemic patients (*p* = 0.02), and anaemia and ID were concomitantly present in 117 (9.8%) of the patients.

**Fig. 1:**
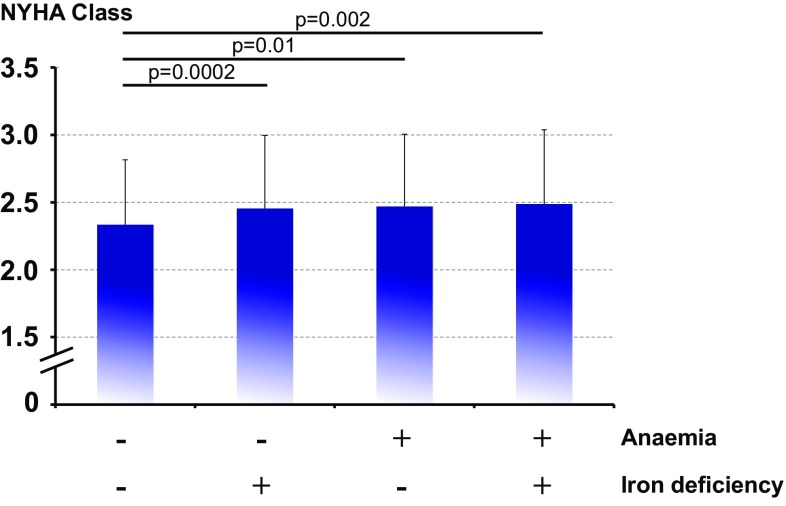
NYHA class according to baseline status being iron-deficient, anaemic, both or none of the two. ANOVA *p* < 0.0001

**Fig. 2:**
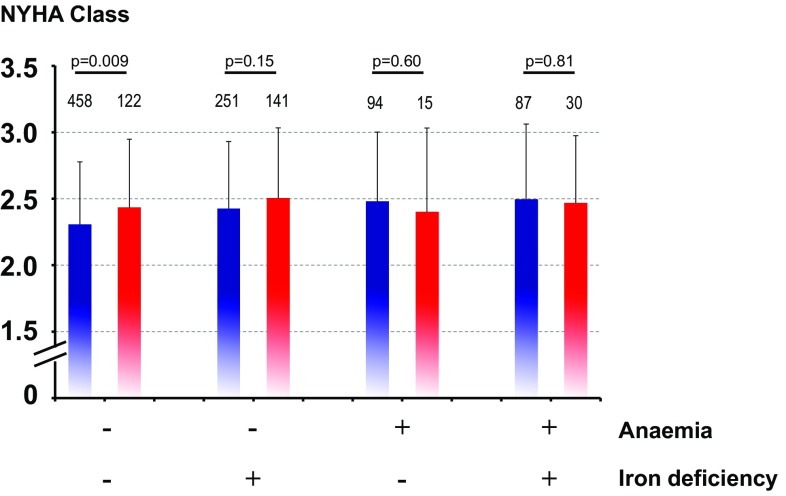
NYHA class according to baseline status being iron-deficient, anaemic, both or none of the two, devided by gender. Male *blue bars*, female *red bars*. Number above the *bars* represents *n* numbers in each group

Baseline haemoglobin strongly correlated with serum ferritin (*r* = 0.16, *p* < 0.0001), TSAT (*r* = 0.26, *p* < 0.0001), C-reactive protein (*r* = −0.15, *p* < 0.0001), and serum creatinine (*r* = −0.14, *p* < 0.0001). When analysed separately, there was a highly significant association between haemoglobin and serum ferritin in patients with ID (*r* = 0.16, *p* = 0.0004), whereas in patients without ID, this association was only of borderline significance (*r* = −0.09, *p* = 0.03). No corresponding associations were noted between haemoglobin and TSAT, C-reactive protein, or serum creatine after splitting the data set into patients with *vs*. without ID. In univariable analysis, the presence of anaemia, ID, or both was associated with significantly higher NYHA class (Figs. 1 and 2).

### Predictors of exercise capacity

Maximum exercise capacity as assessed by spiroergometry was significantly lower in patients with ID (*p* = 0.0004), while regarding the 6-min walking distance, there was no significant difference in patients with versus without ID (*p* = 0.24). Using logistic regression, we found that age, gender, body weight, systolic and diastolic blood pressure, NYHA class, the presence of oedema, LVEF, the presence of anaemia, haemoglobin, the presence of ID, serum ferritin, transferrin saturation (TSAT), serum creatinine, a history of chronic kidney disease, and a history of anaemia all predicted lower exercise capacity (all *p* < 0.05, Table 2). The same was true for BNP or NT-proBNP values above the diagnostic cut-off for non-acute HF (>35 pg/ml and >125 pg/ml, respectively). After adjustment for age, gender, the presence of anaemia, creatinine, LVEF, and natriuretic peptide levels, the presence of ID remained an independent predictor of reduced exercise capacity [odds ratio (OR) 1.323, 95% confidence interval (CI) 1.009–1.735, *p* = 0.04]. Likewise, the presence of anaemia independently predicted reduced exercise capacity after adjusting for age, gender, creatinine, CRP, LVEF, the presence of ID, and natriuretic peptide levels (OR 1.939, 95% CI 1.356–2.773, *p* = 0.0003).

**Table 2:** Univariable and multivariable logistic regression models to predict baseline exercise capacity among 1198 patients with HF with reduced ejection fraction

Variable	Odds ratio (95% CI)	*p*
Univariable models		
Age (1 year increase)	1.085 (1.071–1.100)	<0.0001
Gender (female)	3.788 (2.830–5.072)	<0.0001
Systolic BP (1 mmHg increase)	0.992 (0.986–0.998)	0.007
Diastolic BP (1 mmHg increase)	0.973 (0.962–0.983)	<0.0001
Ischaemic heart disease (present)	1.143 (0.904–1.445)	0.27
NYHA class (1 class increase)	3.246 (2.547–4.136)	<0.0001
LVEF (1 unit increase)	0.968 (0.953–0.984)	<0.0001
Oedema (present)	2.254 (1.699–2.989)	<0.0001
Iron deficiency (present)	1.547 (1.227–1.950)	0.0002
Haemoglobin (1 g/dl increase)	0.693 (0.640–0.751)	<0.0001
Current anaemia (present)	2.361 (1.732–3.219)	<0.0001
History of anaemia (present)	2.597 (1.424–4.736)	0.002
Ferritin (10 µg/l increase)	0.991 (0.985–0.998)	0.007
Log serum ferritin (1 SD increase)	0.789 (0.702–0.887)	<0.0001
TSAT (1 unit increase)	0.985 (0.970–0.999)	0.04
BNP/NT-proBNP above diagnostic cutoff for non-acute HF (present)	2.379 (1.622–3.492)	<0.0001
Log serum creatinine (1 SD increase)	1.323 (1.172–1.493)	<0.0001
Log C-reactive protein (1 SD increase)	1.208 (1.076–1.355)	0.001
History of diabetes mellitus (present)	1.155 (0.901–1.481)	0.26
History of COPD (present)	1.150 (0.771–1.715)	0.49
History of renal dysfunction (present)	2.461 (1.844–3.285)	<0.0001
Multivariable models		
Iron deficiency (present)*	1.323 (1.009–1.735)	0.04
Anaemia (present)**	1.939 (1.356–2.773)	0.0003

*BP* blood pressure, *BNP* B-type natriuretic peptide, *COPD* chronic obstructive pulmonary disease, *LVEF* left ventricular ejection fraction, *NT-proBNP* N-terminal pro-B-type natriuretic peptide, *NYHA* New York Heart Association, *TSAT* transferrin saturation

*Adjusted for age, gender, current anaemia, log serum creatinine, log C-reactive protein, LVEF, and BNP/NT-proBNP above diagnostic cutoff for non-acute HF

**Adjusted for age, gender, log serum creatinine, log C-reactive protein, LVEF, the presence of ID, and BNP/NT-proBNP above diagnostic cutoff for non-acute HF

## Discussion

To our knowledge, the PReP Registry constitutes so far the largest prospective cohort study in ambulatory patients with chronic stable systolic HF evaluating the prevalence and clinical impact of ID and anaemia in a real-world healthcare setting. With 42.5%, ID proved highly prevalent in this population of outpatients taken care of in office-based cardiology practices across Germany. With 18.9% the proportion of anaemia was significantly lower than that of ID. Interestingly, the overlap between the two populations was smaller than one might have expected, since only every second anaemic patient also presented with ID at the same time. Independent of the presence of anaemia, ID predicted lower physical performance irrespective of the exercise test used, i.e., a walking distance or maximum exercise capacity on spiroergometry below the median, which highlights the functional importance of this co-morbidity for the patients. Likewise, the presence of anaemia also predicted a significantly lower maximum exercise capacity.

### Prevalence and possible consequences of iron deficiency

Prevalence and possible consequences of ID complicating HF have more recently been attracting increasing attention. Many (mostly secondary) analyses were published that addressed the prevalence of ID in HF cohorts often derived from other clinical studies [13, 15–17]. In this context, Opasich et al. demonstrated, e.g., that the majority of patients with chronic HF and low haemoglobin levels had anaemia of chronic disease, and nearly, all had deficient iron supply for erythropoiesis and/or blunted endogenous erythropoietin production [15]. Among more than 500 ambulatory patients with stable HF, Jankowska et al. found an ID rate of 32 and 57% in anaemic and non-anaemic subjects, respectively [13]. Schou et al. using the same cut-off criteria as Jankowska et al. to define ID, found among Danish outpatients with characteristics similar to those of the PrEP-participants (25% female, NYHA III-IV, mean LVEF 32%) an ID prevalence of 45% and thus very comparable to our findings [17]. Our findings from the PrEP registry regarding ID prevalence are thus in accordance with published data from several clinical cohorts as well as from smaller outpatient cohorts. The PrEP results also correspond to a recent report from the Studies Investigating Co-morbidities Aggravating Heart Failure (SICA-HF): [18]. Among outpatients with systolic HF, 30% presented with anaemia and 45% with ID. Remarkably, and also in line with our observations, exercise capacity decreased in parallel to decreasing haemoglobin levels (*r* = 0.24, *p* < 0.001) in SICA-HF. Furthermore, exercise capacity proved significantly lower in 19% of the patients who presented with both, anaemia and ID, compared with those with either ID or anaemia, respectively.

Even though the prevalence of restless legs syndrome or a history of depression was low, the proportion of ID among affected PrEP-participants was significantly higher than among patients without ID. This finding is in line with the previous observations by Allen et al., who found a 23.9% prevalence of this syndrome among 251 first-presenters with ID and anaemia and no treatment for restless legs syndrome [19]. A similar association was previously also suggested for depression [20]. In conjunction with the previous findings, our present results indicate the possibility of adverse neuro-psychiatric effects of ID as yet another intriguing facet of its complex systemic sequels. However, prospective research needs to ascertain these interrelations, since our observation of a higher prevalence of patient-reported restless leg syndrome or a history of depression in patients with ID does not establish causality, as it is subject to various kinds of bias. In particular, future studies need to employ appropriate validated tools to evaluate and quantify both conditions at the time of study enrolment rather than rely on patients’ history.

### Therapeutic options in iron deficiency and anaemia

Common clinical practice treatment options of anaemia in patients with HF, which might be considered after exclusion of treatable/reversible causes (e.g., gastrointestinal bleeding), include application of erythropoietin and its derivatives, blood transfusions, or iron administration. However, the Reduction of Events in Heart Failure trial (RED-HF) using darbepoetin-alfa in anaemic patients with HF recently ended neutral, [21] and blood transfusions should be restricted to selected symptomatic patients with very low haemoglobin levels, predominantly those <8 g/dl, leaving repletion of iron stores as the only promising treatment option with anaemia and ID that may be applied routinely in clinical practice. Our findings of a high prevalence of ID not always coincide with anaemia among PrEP-participants and the independent association of ID with reduced exercise capacity in our cohort buttress applicability of the current guideline recommendation regarding repletion of ID also in the outpatient setting. They suggest that iron substitution should be considered in symptomatic outpatients with chronic stable HF with reduced ejection fraction and ID and anaemia, but also, if there is no concomitant anaemia [2].

Using the same definition of ID as employed in the PrEP registry, the Ferinject Assessment in patients with IRon deficiency and chronic Heart Failure (FAIR-HF) trial randomized patients with symptomatic HF and a LVEF ≤40% (or ≤45% and NYHA III) with ID and haemoglobin levels between 9.5 and 13.5 g/dl to intravenous ferric carboxymaltose or saline [22]. After 24 weeks, self-reported patient global assessment had improved significantly more often in patients receiving ferric carboximaltose (50% vs 28%, *p* < 0.001), and various secondary endpoints, including NYHA class and quality of life, had also improved [23]. Using a similar study design, the ferric CarboxymaltOse evaluatioN on perFormance in patients with IRon deficiency in coMbination with chronic Heart Failure (CONFIRM-HF) study extended these findings in an outpatient population with ID and similar characteristics to an observation period of 52 weeks [24]. Importantly, hospital admissions for worsening HF, which constituted a pre-defined secondary endpoint, were significantly reduced in CONFIRM-HF. The Effect of Ferric Carboxymaltose on Exercise Capacity in Patients With Iron Deficiency and Chronic Heart Failure (EFFECT-HF) trial, even though open label, further extended these findings by showing that intravenous iron therapy with ferric carboxymaltose improves exercise capacity as assessed using spiroergometry [25]. A recent meta-analysis involving five trials, which evaluated intravenous iron therapy in iron-deficient patients with systolic heart failure, confirmed that this treatment improves clinical outcomes, exercise capacity, and quality of life, and to alleviate HF symptoms [26]. In line with these results, another meta-analysis showed that iron supplementation reduced the need for blood transfusions and increased haemoglobin levels in patients with chronic kidney disease or in the peri-partal period [27]. The recently published Oral Iron Repletion effects ON Oxygen UpTake in Heart Failure (IRONOUT) trial has underscored missing benefit of oral iron therapy, as no increase in exercise capacity and only a minimal increase in serum ferritin level were noted after 12 weeks of therapy [28].

Against this background, current guidelines for the management of HF advocate the repletion of ID with the inclusion criteria of FAIR-HF as defining features for patients eligible for treatment [2]. In addition, the guidelines state that blood biochemistry parameters should be regularly assessed to “detect reversible/treatable causes of HF (e.g., hypocalcaemia, thyroid dysfunction) and co-morbidities (e.g., iron deficiency)”. With regard to anaemia, the guidelines list “chronic HF, haemodilution, iron loss or poor utilization, renal failure, chronic disease, (and) malignancy” as potential reasons for anaemia development, and recommend to perform a “diagnostic work-up” and to “consider treatment” [2]. A recent French position paper states that iron supplementation should be considered “for all patients with chronic HF or hospitalized for acute decompensation of chronic HF with biological evidence of iron insufficiency” [29].

### Limitations

Findings of this study should be interpreted in the light of its limitations. First, the PrEP registry derived all study data from informations obtained during routine patient care. Patient phaenotyping was, therefore, not strictly standardized and performed according to local circumstances, which precluded, e.g., the use of uniform exercise testing or determination of just one natriuretic peptide. The diagnosis of co-morbidities as restless leg syndrome, depression, diabetes, or COPD was based on patients’ reports and not ascertained in the frame of the study. Moreover, cardiologists were asked to recruit patients consecutively, but did not complete a screening and enrolment log. Although they did not report any patients denying participation, patient selection may have been biased by factors unaccounted for in this study. Finally, PrEP-participants were recruited between 2010 and 2012, when in general, diagnosis and management of co-morbidities received less attention than recommended in the most recent version of the European Society of Cardiology HF guidelines. Awareness of anaemia and ID as common characteristics of the HF syndrome with adverse impact on prognosis may since have increased also amongst physicians responsible for HF outpatient care. However, the PrEP registry results, derived from the so far largest stable outpatient cohort of symptomatic HF patients, provide for the first time strong arguments to diagnose and treat these conditions also in stable outpatients attending cardiologist practices, and appear suited to call cardiologists into responsibility in this respect. PrEP results may thus significantly reinforce routine assessment of iron status and help to establish iron supplementation as a treatment option also in current ambulatory HF care.

### Conclusions

The PReP registry, which involved a large cohort of patients with symptomatisc systolic HF enrolled in 42 German office-based cardiology practices, demonstrated high prevalence of both anaemia and ID (18.9% and 42.5%, respectively) in this population. Despite this high prevalence, anaemia was often unappreciated, and ID was previously unknown in all PrEP-participants. Clinical relevance, treatability, and independent association with reduced physical performance as demonstrated by the PrEP results call for routine diagnostic clarification of anaemia and ID-screening in ambulatory healthcare settings to further improve current outpatient HF care.
